# Bovine Pre-adipocyte Adipogenesis Is Regulated by bta-miR-150 Through mTOR Signaling

**DOI:** 10.3389/fgene.2021.636550

**Published:** 2021-02-03

**Authors:** Xingyi Chen, Sayed Haidar Abbas Raza, Xinhao Ma, Jiangfang Wang, Xiaohui Wang, Chengcheng Liang, Xinran Yang, Chugang Mei, Syed Muhammad Suhail, Linsen Zan

**Affiliations:** ^1^College of Animal Science and Technology, Northwest A&F University, Xianyang, China; ^2^Department of Livestock Management, Breeding and Genetics, The University of Agriculture, Peshawar, Pakistan; ^3^National Beef Cattle Improvement Center, Northwest A&F University, Xianyang, China

**Keywords:** bta-miR-150, mTOR, RNA-seq, adipocyte differentiation, WGCNA

## Abstract

Micro RNA (miR) are recognized for their important roles in biological processes, particularly in regulatory componentization. Among the miR, miR-150 has been the focus of intense scrutiny, mostly due to its role in malignant tumors. A comparison between steer and bull adipose tissues identified bta-miR-150 as one of the nine downregulated miRNAs, although its function remains unknown (GEO:GSE75063). The present study aimed to further characterize the role of bta-miR-150 in cattle. bta-miR-150 has a negative regulatory effect on the differentiation of bovine adipocytes and promotes proliferation. Overexpression of bta-miR-150 can promote mRNA and protein expression of the marker genes *CDK1, CDK2*, and *PCNA*, increase the number of EdU-stained cells, promote adipocyte proliferation, inhibit adipocyte differentiation, and reduce lipid droplet formation. Results of RNA-seq and WGCNA analyses showed that the mammalian target of the rapamycin signaling pathway, which plays a major regulatory role, is dysregulated by the overexpression and inhibition of miR-150. We found that the target gene of bta-miR-150 is *AKT1* and that bta-miR-150 affects *AKT1* phosphorylation levels. These results showed that bta-miR-150 plays a role in adipogenic differentiation and might therefore have applications in the beef industry.

## Introduction

Adipose tissue that differentiates from mesenchymal stem cells during the embryonic period is responsible for many functions, including energy storage, lipid metabolism, and hormone secretion (Ambele et al., 2020; Poklukar et al., 2020). Since the amount of adipose tissue in meat impacts the economic value of animals, understanding the molecular mechanisms underlying adipose tissue generation in animals will ensure that production is maximized within an appropriate range. MicroRNA are non-coding RNA with a maturity length of 18–25 nt; they are ubiquitous in the animal kingdom and are produced by RNA polymerase II transcription. MicroRNA are involved in many different physiological processes in animals (Raza et al., 2020), including inhibition of the translation of target genes, affecting mRNA degradation, and regulating the transcription of target genes (Twayana et al., 2013). Differentiation of adipocytes is regulated by many miRNA, which are the focus of the current investigation. Advancements to high-throughput sequencing technology has allowed deeper exploration of cellular regulatory processes (Katz et al., 2010). Previous studies have already revealed the regulatory effects of human miRNA, including miR-204 (Zhang Z. et al., 2020), miR-27a (Kim et al., 2010), miR-210 (Ren et al., 2020), miR-24 (Liu et al., 2020), miR-378 (Duarte et al., 2020), miR-149-5p (Khan et al., 2020), miR-143 (Zhang L. et al., 2020), and miR-145 (Wang et al., 2020), on adipogenesis. These classic mechanisms also occur in the adipose tissue, where they have a large impact on the surrounding tissue (Arcidiacono et al., 2020; Raza et al., 2020). For example, miR-143 promotes the differentiation of adipocytes by directly acting on its target gene, *MAP2K5*. Many biologically significant miRNA have been discovered, with high-throughput sequencing technology continuously improving in parallel with a reduction in cost (An et al., 2016).

Because of its high expression level in immune-related cells, hsa-miR-150 was one of the first miRNAs to be studied in humans. Human (hsa)-miR-150 plays an important role in the differentiation of hematopoietic cell lines; thus, investigations of hsa-miR-150 have mainly focused on malignant tumors (Yugawa et al., 2020). In animal, miR-150 has been implicated in the mTOR pathway, where it regulates the expression of leptin in adipocytes (Scrutinio et al., 2017) and the production of lipids such as triglycerides (TGs) and free fatty acids (FFAs). The master metabolic factor PGC-1a mediates the reciprocating cycle between TG and FFA to achieve physiologically stable energy consumption (Kang et al., 2018). In pigs, ssc-miR-150 is differentially regulated in the adipose tissue miRNA of lean, rather than fatty pig breeds. Ssc-miR-150 is thought to act directly on the 3′UTR of *CYP3A4* to promote FFA-induced adipose tissue degeneration (Ma et al., 2020). Since miRNA is highly conserved in the animal kingdom, bta-miR-150 might also function in the production of adipose tissue in cattle. Importantly, bta-miR-150 is one of the nine downregulated miRNA among differential expression profiles of steer and bull fat tissues (Liang et al., 2019).

With the global increase in human population and improvements in living standards, beef fat is increasingly being valued by breeders for the flavor it imparts, as well as its high energy value as a food source (Lillehammer et al., 2011). The 480-amino-acid enzyme, RAC-alpha serine/threonine-protein kinase (AKT1), located downstream of the mammalian target of the rapamycin signaling (mTOR) pathway regulates the growth of mammalian cells and is an important protein for regulating fat deposition (Zhang et al., 2017; Song et al., 2018). In mice, miR-150 inhibits *AKT1*. Bovine chromosome 21 encodes *AKT1*, which is an important member of the PI3K/AKT/mTOR pathway. The activity of *AKT* requires phosphorylation at Thr308 and is further enhanced by phosphorylation at Ser473. When Ser473 is simultaneously phosphorylated by *PDK1* and *mTORc2, AKT1* further regulates cell proliferation through downstream FOXO transcription factors and p53 regulatory factors (Cai et al., 2019; Sanchez-Gurmaches et al., 2019; Du et al., 2020) The present study aimed to determine the relationship between bta-miR-150 and *AKT1* by examining the effects and mechanisms of bta-miR-150 in regulating the proliferation and differentiation of Qinchuan beef cattle pre-adipocytes, thus providing a basis for targeted breeding and genetic improvement based on bta-miR-150. In beef cattle, the process of breeding to increase the intramuscular fat content would be supported by an understanding of the genetic basis of adipogenesis. Our research further clarifies the relationship between bta-miR-150 and adipocyte differentiation in cattle. This information paves the way for further research to expand our understanding of the complex process of adipocyte differentiation and adipose tissue generation.

## Materials and Methods

### Isolation of Primary Bovine Pre-adipocytes and Cell Culture

The tissue and cell samples used in the experiment in this study were collected from three 1-day-old healthy Qinchuan cattle bulls with consistent growth bred from the National Beef Cattle Improvement Center of Northwest Agriculture and Forestry University (Yangling, China). Bacteria surgical instruments collected tissue samples such as heart, liver, spleen, lung, and muscle. The sample was placed in a sterile, DNase- and RNase-free cell cryotube. Then the sample was immediately put in liquid nitrogen for freezing, and finally stored in a refrigerator at −80°C for later use.

We used 1-day-old healthy bulls to isolate the original bovine pre-adipocytes, removed the adipose tissue from different parts under aseptic conditions, washed with PBS supplemented with 10% antibiotics (penicillin/streptomycin) 3 times, and then added I Collagenase digestion for 1–2 h in a 37°C water bath shaker. After digestion, the cells were filtered with a cell sieve and the supernatant was discarded after centrifugation. The red blood cell lysate was added for lysis, and then an appropriate amount of DMEM-F12 (Gibco, Grand Island, NY) medium containing 10% fetal bovine serum (FBS, Invitrogen) was added to resuspend the cells for seeding for subsequent experiments. The cells used in this experiment were all the 3rd generation cells after passage.

### Construction and Transfection of Plasmid and RNA Oligonucleotides

The cells were inoculated in DMEM-F12 supplemented with 10% FBS and 1% antibiotics and kept continuously at 37°C and 5% CO_2_. According to the manufacturer's instructions, the miR-150 mimic (50 nM), miR-150 mimic NC (50 nm), miR-150 inhibitor (100 nM), miR-150 inhibitor NC (100 nM), si-*AKT1* (100 nM), and si-NC were transfected into the cells using Lipofectamine^TM^ 3000 (Invitrogen, San Diego, GA, LUSA). We induced differentiation of adipocytes 48 h after transfection. After 2 days of induction with DMI (0.5 mm IBMX, 1 μm DXMS, and 2 μm insulin) induction solution, we changed to a maintenance medium containing 5 μg/mL insulin. After differentiation induction, adipocytes were harvested at d0, d2, d4, d6, d8, and d10. (We recorded the day of adding DMI as day 0 of induction of differentiation). Two restriction enzymes (Takara, Beijing, China), XhoI and NotI, were used to construct the vector psiCHECK-2(Laboratory retention). The detailed sequence is shown in Table 1. The Bta-miR-150 mimic, mimic NC, inhibitor, and inhibitor NC used in this stage were purchased from RiboBio, Guangzhou, China; the si-*AKT1* and si-NC used in this stage were purchased from GenePharma, Shanghai, China. The detailed sequence in is shown Table 1.

**Table 1:** The sequence of RNA oligonucleotides and plasmid.

**Name**	**Sequence (5′-3′)**
miR-150 mimic	sense: UCUCCCAACCCUUGUACCAGUGU antisense: ACACUGGTACAAGGGUUGGGAGA
mimic NC	UUGUACUACACAAAAGUACUG
miR-150 inhibitor	ACACUGGUACAAGGGUUGGGAGA
inhibitor NC	CAGUACUUUUGUGUAGUACAA
si-AKT1	sense: GCCAUGAAGAUCCUAAAGATT antisense: UCUUUAGGAUCUUCAUGGCTT
si-NC	sense: UUCUCCGAACGUGCACGUTT antisense: ACGUGACACGUUCGGAGAATT
psiCHECK-2	5′… GAGCAGTAATTCTAGGCGATCGCTCGAG CCCGGGAATTCGTTTAAACCTAGAGCGGCCGCTGGCCGC AATAAAATA… 3′

### Differential Expression and Pathway Enrichment Analyses

Transfected miR-150 mimic, mimic NC, inhibitor, inhibitor NC, and bovine adipocytes were used to induce differentiation, and RNA on day 2 and day 6 were collected. A control was set for each treatment, with three technical replicates per group and a total of eight groups (24 samples). After RNA extraction, the HiSeq-PE150 platform (Illumina, Sandiego, CA, USA) was used for RNA sequence analysis. After filtering the original data, the reads were matched to the bovine genome using HiSat2 (Bos-taurusUMD3.1, release94, Ensembl database), and feature counts were used to count gene expression. The DESeq2 R software package was used to screen DEGs, with Log_2_ fold change (log_2_FC) > 1 and false discovery rate (FDR) < 0.05 as the screening criteria. ClusterProfiler was used to realize KEGG pathway analysis and FDR < 0.05 was considered significant.

### Extraction of RNA, Quantitative Real-Time PCR (qRT-PCR)

After 48 h of transfection, we used the RNAiso Plus Kit (Trizol, Takara, Beijing, China) to extract RNA. The specific extraction method can refer to the reference (Junjvlieke et al., 2020). After we removed the culture medium from the cells, we washed the cells with PBS 2~3 times in each well, then added 1 mL Trizol, placed the mixture at 4° for 4 min, and transferred the mixture to a 1.5 mL centrifuge tube. Two hundred microliters of chloroform was added to the centrifuge tube, shaken vigorously for 30 s, left to stand for 5 min, and centrifuged for 15 min. The supernatant was then taken (3 layers in total), isopropanol was added, mixed upside down, left to stand on ice for 10 min, and then centrifuged for 10 min. After centrifugation, add 1 mL of 75% cold ethanol was slowly added to a small amount of precipitation at the bottom, centrifuged at 4°C for 5 min, and then the ethanol was discarded. The cells were dried for 2–5 min at room temperature, then an appropriate amount of RNase-free DEPC water was added to dissolve the precipitate. NanoQuant plateTM (TECAN, Infinite M200 PRO) was used to determine the concentration and purity of RNA, and it was stored at −80°C for later use. The method of extracting total RNA in tissues was to take out about 0.5 g tissue sample in a refrigerator at −80°C, add it to liquid nitrogen for pre-cooling, and then place it in a mortar cooled with liquid nitrogen for grinding. After grinding, the sample is transferred to a 1.5 mL enzyme-free centrifuge tube with 1 mL Trizol, and then placed on ice for 15 min. The next step is the same as that of extracting cellular RNA.

Reverse transcription of total RNA was performed following Prime Script ™ RT reagent Kit with gDNA Eraser (Takara, China), TB Green^®^Premix Ex TaqTM II(TAKARA, Beijing, China) instructions. The reverse transcription and qPCR of miRNA were performed with miRcute Plus miRNA First-Strand cDNA Kit and miRNA Plus miRNA qPCR Kit (SYBR Green) (Tiangen, Beijing, China). U6 and β – actin was used as the internal control for miRNA quantification and mRNA quantification, respectively. The sequence of all PCR primers is shown in Table 2. All experiments were performed with three biological replicates and three technical replicates. The 2-ΔΔCt method was used to analyze the relative expression levels of different qPT-PCR data.

**Table 2:** mRNA and miRNA Real-time quantitative PCR primer sequences.

**Genes**	**Primer Sequence (5′-3′)**	**Annealing**
		**temperature**
β-actin	F: CATCGGCAATGAGCGGTTCC R: CCGTGTTGGCGTAGAGGTC	60°C
PCNA	F: CCTTGGTGCAGCTAACCCTT R: TTGGACATGCTGGTGAGGTT	60°C
CDK1	F:AGTGGAAACCAGGAAGCTTAG R:ATTCGTTTGGCAGGATCATAGA	60°C
CDK2	F:GGGTCCCTGTTCGTACTTATAC R:CCACTGCTGTGGAGTAGTATTT	60°C
AKT-1	F:AAGGAGATCATGCAGCACCGATTC R: GTCTTGGTCAGGTGGCGTAATGG	60°C
CEBPα	F: ATCTGCGAACACGAGACG R: CCAGGAACTCGTCGTTGAA	60°C
PPARγ	F: GACGACAGACAAATCACCGT R: CTTCCACGGAGCGAAACTGA	60°C
FABP4	F:TGAGATTTCCTTCAAATTGGG R: CTTGTACCAGAGCACCTTCATC	60°C
RRAGD	F: TAAGAAGGAGCGGCAAGT R: GGAAGTCCCAAATCTGAAA	60°C
RPS6KA3	F:ACCTAGCAACATTCTTTATGTGGAT R: GCATCATAGCCTTGTCGT	60°C
RPS6KB1	F: ATCACCAAGGTCACGTCAAAC R: TGCTCCCAAACTCCACCAAT	60°C
LPIN1	F: AGTGAATCTTCAGATGCGTTTA R: CAGGTGTCGGCTTCGTTT	60°C
NFKB1	F: GAGAACTTCGAGCCTCTGTAC R: CTCATAGGGTTTCCCATTTA	60°C
EIF4E	F: TCTAATCAGGAGGTTGCT R: CCCACATAGGCTCAATAC	60°C
PIK3CA	F:TGGGTTTCTCGGTCCTAA R:CAGTCCGTCCAGTCATCC	60°C
TNFRSF1B	F: CCAGCACAGCTCCAAGCA R:CAACTATCAGAAGCAGACCCAAT	60°C
INSR	F: GGAAGGCGAGAAGACCAT R: TGACACCAGGGCATAGGA	60°C
EIF4EBP2	F: GTTCCTGATGGAGTGTCGGA R: AACTGTGACTCTTCACCGCCT	60°C
PIK3R1	F: GAGCGGGAAGAGGACATTGA R: TCTCCCCTGTCGTCTCGTTA	60°C
mTOR	F: TGATGCAGAAGGTCGAGGTG R: GATGGGTGTCTATCGCCCAG	60°C
PIK3R1	F: CTATCTCCTGGACTTACCG R: GGCGACCTAATGAGTTTC	60°C
U6	F:TAGCCACCCTCAAGTATGTTCG R:CGAGGTAGGAGGACAGGAGT	60°C
miR-150	UCUCCCAACCCUUGUACCAGUGU	60°C

### Western Blot Analysis

The methods of extraction and Western blot analysis of total protein have been introduced in references (Khan et al., 2019; Wang et al., 2019). The protein bands added with Millipore chemiluminescence solution were placed on the Gel DocTMXR+ System (Bio-Rad, Hercules, CA) for exposure imaging. The antibody in Table 3 is the antibody we used in the experiment.

**Table 3:** Antibody information.

**Name of antibody**	**Immune features**	**The company**	**Dilution ratio**
Anti-AKT1 antibody	Rabbit polyclonal	CST	1:1,000
Phospho-Akt (Ser473) antibody	Rabbit polyclonal	CST	1:1,000
Anti-CDK1 antibody	Rabbit monoclonal	Abcam	1:1,000
Anti-CDK2 antibody	Rabbit monoclonal	Abcam	1:1,000
Anti-PCNA antibody	Rabbit monoclonal	Abcam	1:5,000
Anti-PPARγ antibody	Rabbit polyclonal	Boster	1:1,000
Anti-FABP4 antibody	Rabbit monoclonal	Abcam	1:1,000
Anti-CEBPα antibody	Rabbit monoclonal	Abcam	1:1,000
Anti-β-actin antibody	Rabbit polyclonal	Abcam	1:10,000

### EdU Staining and CCK-8 Assay

The Qinchuan cattle pre-adipocytes were seeded in a 96-well plate (NEST, Jiang Su, China) and cultured to a density of 40–60% confluence, which was reached at a density of 5.0 × 10^4^ cells for transfection. The specific processing steps are detailed in reference (Chu et al., 2019). We added 100 μL PBS to each well of the last stained cells and washed them 1 to 3 times, and then took photographs under a microscope. The instrument used for taking pictures was an Olympus 1 × 71 microscope (Olympus, Tokyo, Japan). The treatment method of CCK-8 cell cycle detection was the same as that of EdU. The specific operation steps were carried out according to the reference (Khan et al., 2020). The kit used was TransDetect Cell Counting Kit (Transgen biotechnology, Beijing, China), and the detection instrument was an Infinite^®^200PRO (Tecan Trading AG, Switzerland).

### Bodipy, Oil Red O Staining, and Triacylglycerol Assay

The differentiated cells were stained for different days. The cells on the second day were stained with Bodipy, and the cells on the fourth and sixth days were stained with oil red O. In short, both Bodipy (1:1,000) and DAPI (1:1,000) were diluted in PBS. The cells to be stained were washed with PBS 3 times, fixed with 4% paraformaldehyde for 30 min, and then stained with Bodipy for 30 min. After staining, the cells were washed with PBS 3 times and then stained with DAPI. Finally, the cells were photographed under a microscope. The whole process needs to be done away from light.

The oil red O staining procedure was as follows: the cells were washed in the six-well plate with PBS 3 times, fixed with 4% paraformaldehyde for 30 min, dyed with 60% oil red O (solvent: isopropanol, 0.15 g oil red powder/100 mL) for 30 min, then washed with PBS and observed under a microscope. We measured the amount of intracellular TAG using the cell/tissue triglyceride assay kit (Applygen Technologies, Beijing, China) according to the protocol recommended by the manufacturer (Ma et al., 2018).

### Luciferase Reporter Assay

To verify whether bta-miR-150 targets the 3′-UTR of AKT1, we obtained the mature sequence of bta-miR-150 from the miRBase (http://www.mirbase.org/). The reporter of luciferase was produced by ShengGong (Sangon Biotech Co., Ltd, Shanghai, China) through synthesizing 3′-UTR of AKT1 wild-type (WT) and mutation-type (MUT) of AKT1 sequence. Xhol and Notl cutting sites of Wt-3′-UTR containing miR-150 target were amplified. AKT1-3′UTR WT or MUT were cloned into psiCHECK-2 vector (Laboratory retention) by Xhol and Notl restriction sites (Promega, Madison, WI, USA). After culturing HEK293A cells for 2 days, they were then co-transfected into bovine adipocytes with about 0.16 μg of MUT vector/AKT1-3′ UTR WT and 5 pmol miR-150 mimic/negative control. At 48 h after transfection, the luciferase activity was measured by Dual-Luciferase^®^ Reporter Assay System (Promega, Madison, WI, USA). Luciferase assay was carried out in three repeat wells, and three experiments were carried out.

### Weighted Gene Co-expression Network Analysis (WGCNA)

WGCNA, an R package, was used to construct the weighted gene co-expression network. With the standardized gene expression matrix as input, the variation degree of each gene expression level among samples was calculated, and the top 50% genes with the largest variation were selected for WGCNA. After the threshold screening, the scaleless adjacency matrix was obtained by power processing with β = 12. In order to better evaluate the correlation of gene expression patterns, the adjacency matrix was further transformed into a topological overlap matrix (ToM), and the dynamic cutting algorithm was used to cluster and partition genes by using the topological difference matrix (distom = 1-tom).

### Annotation of Gene Modules and KEGG Analysis

In order to explore the biological function of gene modules, David and KOBAS online tools were used to analyze the gene go and KEGG enrichment to describe the function of the modules and identify the relationship between these modules. The *p*-value was adjusted by the Benjamin Hochberg method.

### Statistical Analysis

Data are expressed as mean ± SD. Statistical analysis was performed using SPSS 19.0 software (SPSS, Chicago, IL, USA) and GraphPad Prism 6. One-way ANOVA and Dunnett's multiple comparison test were used to analyze *P*-values. Student's *t*-test was used for comparative analysis of the two groups, and *p < 0.05, **p < 0.01, ***p < 0.001 were considered statistically significant.

## Results

### miR-150 Is Up-Regulated in the Adipose Tissue From Qinchuan Cattle

**Figure 1:**
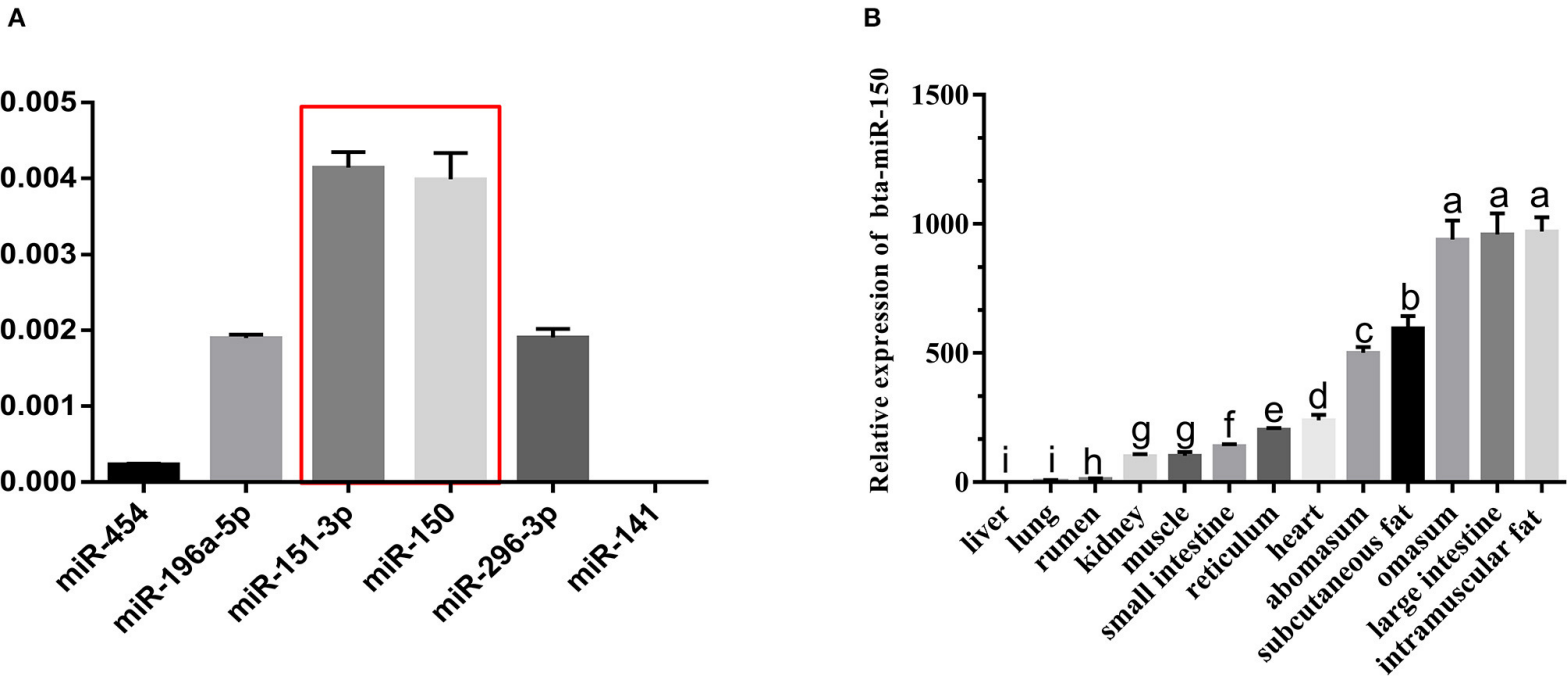
miR-150 is expressed at high levels in bovine adipocytes. **(A)** Background expression of miR-150. **(B)** Tissue expression profile of miR-150. Values with superscript letters indicate mean significant difference (*p* < 0.05).

To initially screen for candidate miRNA that regulate the development of bovine adipose tissue, we selected 52 differentially expressed (DE) miRNA (log_2_(FC) > 1, FDR < 0.05) based on our previous sequencing of transcriptomes from steers and bulls (GEO:GSE75063). Among these, 16 DE miRNA with sequence homology to human miRNA and their target DE genes (DEG) were assessed using Ingenuity Pathway Analysis (IPA) to construct a gene interaction network. In total, nine of these DE miRNA and 42 target DEG were mapped to a network that is involved in the differentiation of fat cells and fat tissue metabolism (Zhang et al., 2017). We tested background levels of miRNA with unknown or incomplete functional annotation by qPCR and found that background levels of miR-150 and miR-151-3p were higher than those of other miRNA in fatty tissues (Figure 1A), which was consistent with previous (Zhang et al., 2017). We therefore speculated that miR-150 is involved in the development and regulation of bovine adipose tissue (Figure 1B).

### Bovine Pre-adipocyte Proliferation Is Promoted by miR-150

**Figure 2:**
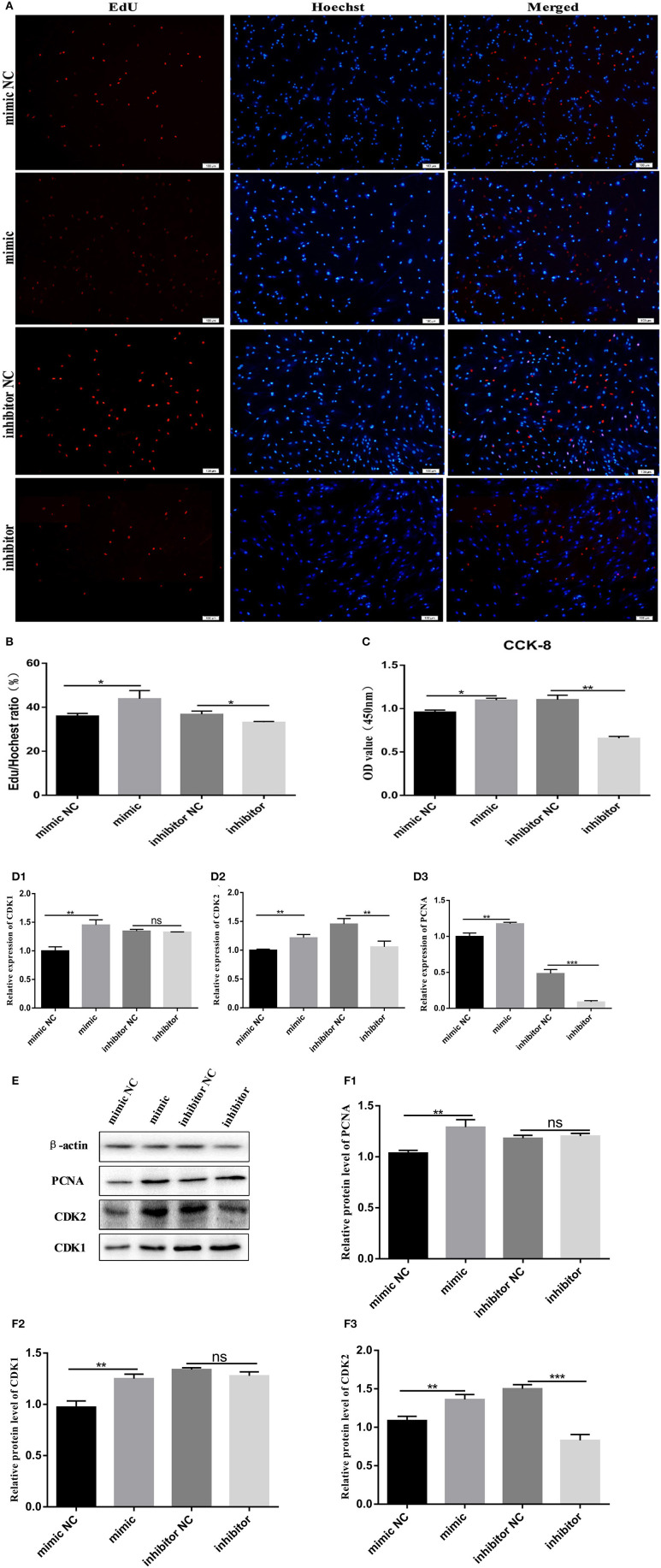
Proliferation of Qinchuan bovine pre-adipocytes is promoted by bta-miR-150. **(A,B)** Bovine adipocytes transfected with mimic, mimic NC, inhibitor, and inhibitor NC. Cells harvested at 48 h after transfection were stained red with EdU (to indicate DNA replication) and blue with Hoechst (cell nuclei) **(C)** CCK-8 assays of bovine adipocytes. After incubating with 10% CCK-8 for 4 h, absorbance was measured at a wavelength of 450 nm. **(D1–D3)** Changes in mRNA levels of proliferation-related target genes according to qPCR findings. **(E, F1–F3)** Proliferation-related protein quantitation according to western blot findings. Error bars represent mean ± SD. *N* = 3 replicates. *Significantly different; *p < 0.05, **p < 0.01, ***p < 0.001, Student *t*-tests.

We transfected bovine pre-adipocytes with a miR-150 mimic, mimic NC, miR-150 inhibitor, and inhibitor NC to further explore the effects of miR-150 on the proliferation of bovine pre-adipocytes. We initially stained cells with EdU. At 48 h after transfection, the rate of EdU-positive cells was significantly higher among pre-adipocytes incubated with the bta-miR-150 mimic than with mimic NC (*p* < 0.05), and the rate of EdU-positive cells was significantly lower in cells transfected with bta-miR-150 inhibitor than with inhibitor NC (*p* < 0.05) (Figures 2A,B). The results of CCK-8 assays were consistent with these findings (Figure 2C).

We then assessed changes in expression levels of proliferation marker genes at the mRNA and protein levels. Bovine pre-adipocytes were incubated for 48 h with mimic and inhibitor miR-150, and then, total RNA was extracted. The expression levels of genes associated with the cell cycle, such as *CDK1* (cyclin dependent kinase1), *CDK2* (cyclin dependent kinase2), and *PCNA* (proliferating cell nuclear antigen) significantly increased in cells incubated with the mimic and significantly decreased in those incubated with the inhibitor (Figures 2D1–D3) compared with that in the negative control. Western blotting showed that proliferating pre-adipocytes incubated with mimic miR-150 had significantly increased protein levels of CDK1, CDK2, and PCNA, while those incubated with miR-150 inhibitor had significantly reduced protein levels than the negative control (Figures 2E,F1–F3). These findings suggest that miR-150 promotes bovine pre-adipocyte proliferation.

### Inhibition of miR150 Promotes Adipogenesis

**Figure 3:**
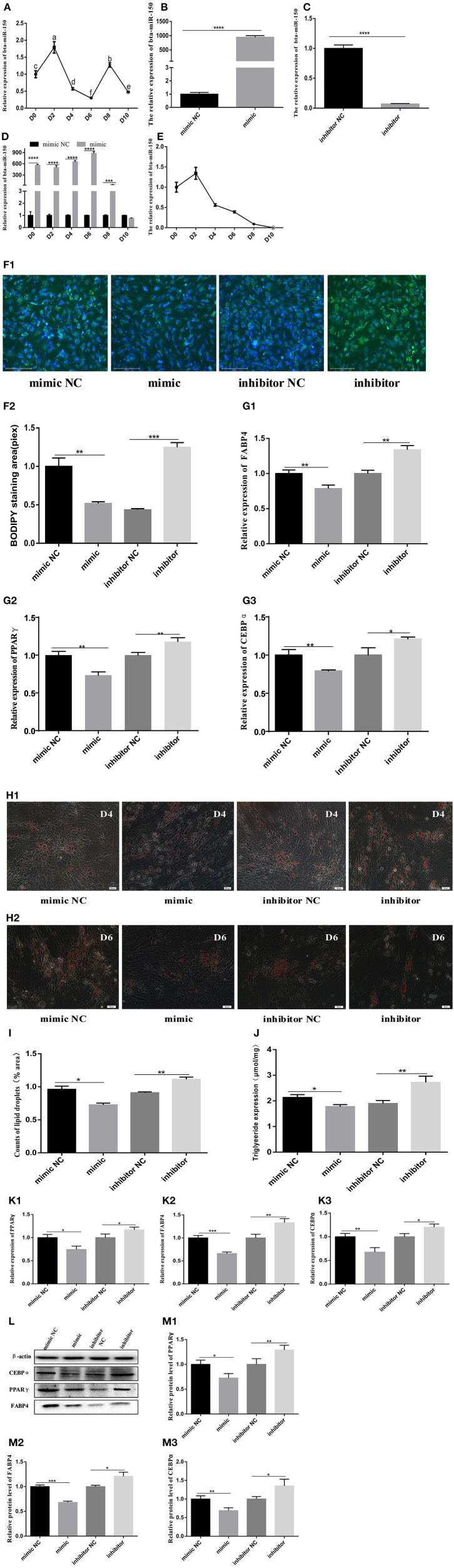
Differentiation of pre-adipocytes from Qinchuan cattle. **(A)** Background expression of bta-miR-150 on different days after inducting differentiation in Qinchuan cattle adipocytes. **(B)** bta-miR-150 mimic transfection efficiency. **(C)** bta-miR-150 inhibitor transfection efficiency. **(D)** Overexpression of bta-miR-150 treated with miR-150 mimic in 10 days. **(E)** Changes of bta-miR-150 content in Qinchuan cattle adipocytes treated with miR-150 mimics. **(F1,F2)** After transfection of miR-150 mimic, miR-150 inhibitor, or negative control, the adipocytes were stained with Bodipy and quantified on the second day of differentiation; photos were taken with an Evos-fl-auto2 automatic living cell fluorescence microscopy imaging system (Thermo Fisher, USA). **(G1–G3)** Changes in mRNA levels of adipose differentiation marker genes on the second day of differentiation (FABP4, PPARγ, and CEBPα). **(H1)** Oil red O staining of mimics, inhibitors, and their respective internal controls on day 4; the photos were taken with an Olympus Image IX71 microscope (Olympus, Japan). **(H2)** bta-miR-150 mimic treatment on day 6, Oil red O staining of substances, inhibitors, and their respective internal controls; the photos were taken with an Olympus Image IX71 microscope (Olympus, Japan). **(I)** Statistics of lipid droplet content on day 4 of differentiation, using Image J software. **(J)** Triglyceride content on day 4 of differentiation. **(K1–K3**) Changes in mRNA levels of fat differentiation marker genes (FABP4, PPARγ, CEBPα) on the 4th day. **(L)** Protein map of fat differentiation marker genes (FABP4, PPARγ, CEBPα). **(M1–M3)** Quantitative processing of fat differentiation marker gene protein results (FABP4, PPARγ, CEBPα); protein levels were quantified and analyzed by Image J software. Error bars represent mean ± SD. *n* = 3 replicates. *denotes significance according to Student's *t*-test, *p < 0.05, **p < 0.01, ***p < 0.001, and ****p < 0.0001.

Total RNA extracted from bovine normal adipocyte cells at 0, 2, 4, 6, 8, and 10 days of induced differentiation *in vitro*, was reverse transcribed into cDNA. Quantitative PCR findings showed that miR-150 mRNA reached the highest level on day 2 of induced differentiation *in vitro*, decreased to the lowest level on day 6, started to increase briefly on day 8, and then began to decline again (Figure 3A). Cells at ~80% density were transfected, and transfection efficiency was measured 48 h later (Figures 3B,C). The effect of the bta-miR-150 mimic was prevalent until day 8 after transfection, but the bta-miR-150 content briefly reached a maximum on day 2 and then continuously fell (Figures 3D,E). We directly assessed the effects of miR-150 on adipose differentiation by staining lipid droplets using BODIPY and oil red O (Figures 3F1,F2,H1,H2). Compared with the control group, the number of lipid droplets in bta-miR-150 mimic-treated adipocytes decreased, while the number of lipid droplets significantly increased in adipocytes incubated with the inhibitor compared with the control. Overexpressed bta-miR-150 significantly inhibited the TG production, which increased when bta-miR-150 was inhibited (Figure 3J). The mRNA and protein levels of peroxisome proliferator-activated receptor gamma (*PPAR*γ), fatty acid binding protein 4 (*FABP4)*, and CCAAT enhancer binding protein alpha (*CEBP*α) were higher with miR-150 inhibition compared with the levels with miR-150 overexpression NC (Figures 3G1–G3,K1–K3). Inhibition of bta-miR-150 therefore promotes adipogenesis.

### GO and KEGG Analysis of Bovine Adipocyte DEG

**Figure 4:**
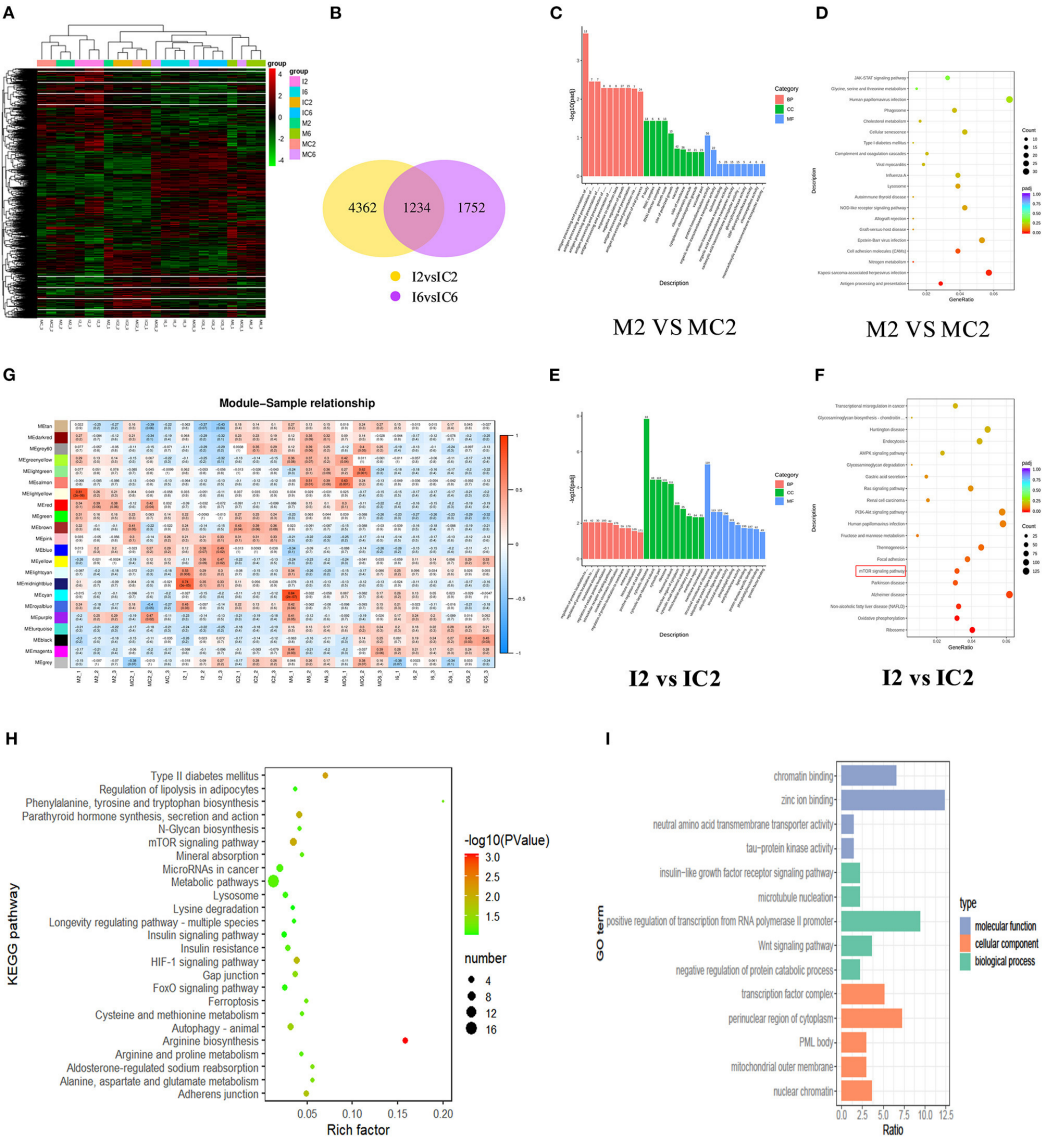
GO and KEGG analysis of bovine adipocytes DEG and WGCNA analysis. **(A)** Differentially expressed gene clustering heat map. The abscissa is the sample name, and the ordinate is the normalized value of the differential gene FPKM. Red color indicates higher expression levels, while green represents lower expression levels; **(B)** The DEGs represented by a Venn diagram. Venn diagram showing the overlapping portion by sharing the expression pattern of the DEGs; **(C)** GO enrichment analysis of mimic treatment group and mimic NC group on the second day; **(D)** KEGG pathway enrichment analysis of mimic treatment group and mimic NC group on the second day;**(E)** GO enrichment analysis of inhibitor treatment group and inhibitor NC group on the second day; **(F)** KEGG pathway enrichment analysis of inhibitor treatment group and inhibitor NC group on the second day; **(G)** Correlation thermogram between samples and modules; **(H)** KEGG enrichment analysis of Midnightblue modules; **(I)** GO enrichment analysis of Midnightblue modules.

We further clarified the regulatory effect of miR-150 on the differentiation of bovine pre-adipocytes using RNA-seq. The miR-150 mimic, mimic NC, inhibitor, and inhibitor NC were transfected into bovine pre-adipocytes as described above, and cells were collected on days 2 and 6 after differentiation. The cells were then incubated daily with miR-150 mimic, mimic NC, inhibitor, and inhibitor NC. Transcriptomes from eight groups (*n* = 3 replicates per group) were sequenced (Beijing Novo Gene, Beijing, China) (Figure 4A). The RNA-seq results of cells transfected with miR-150 mimic, mimic NC, inhibitor, and inhibitor NC of D2, in which a total of 7,348 DEGs were detected, showed there were 1,234 common DEGs in four groups (Figure 4B). We identified the DEG in each group, then assessed them with the KEGG pathway and GO function enrichment analyses and WGCNA analysis (Figures 4C–F).

The soft threshold in the WGCNA analysis was set to 12, and 23 gene modules were detected (Figure 4G). By comparing different groups within the same modules, we identified five modules (green-yellow, light yellow, brown, blue, midnight blue). To better understand the biological function of specific gene modules related to the bovine adipocyte differentiation, we searched for DEG in these five modules using DAVID and KOBAS software using the key words, “fat fractionation” and “metabolism.” We found enrichment for type II diabetes mellitus (ID: bta04930), the mTOR signaling pathway (bta04150), fatty acid degradation (ID: bta00071), and fatty acid metabolism (ID: bta001212) (Figures 4H,I). These pathways are all involved in the biological process of adipogenesis. We combined the RNA-seq data with the WGCNA analysis of KEGG findings and comprehensively integrated *P*-values, numbers of genes changed in the pathway, and the mTOR pathway as main research channels (*P* < 0.05).

### The mTOR Signaling Pathway Is Regulated by bta-miR-150 Through AKT1

**Figure 5:**
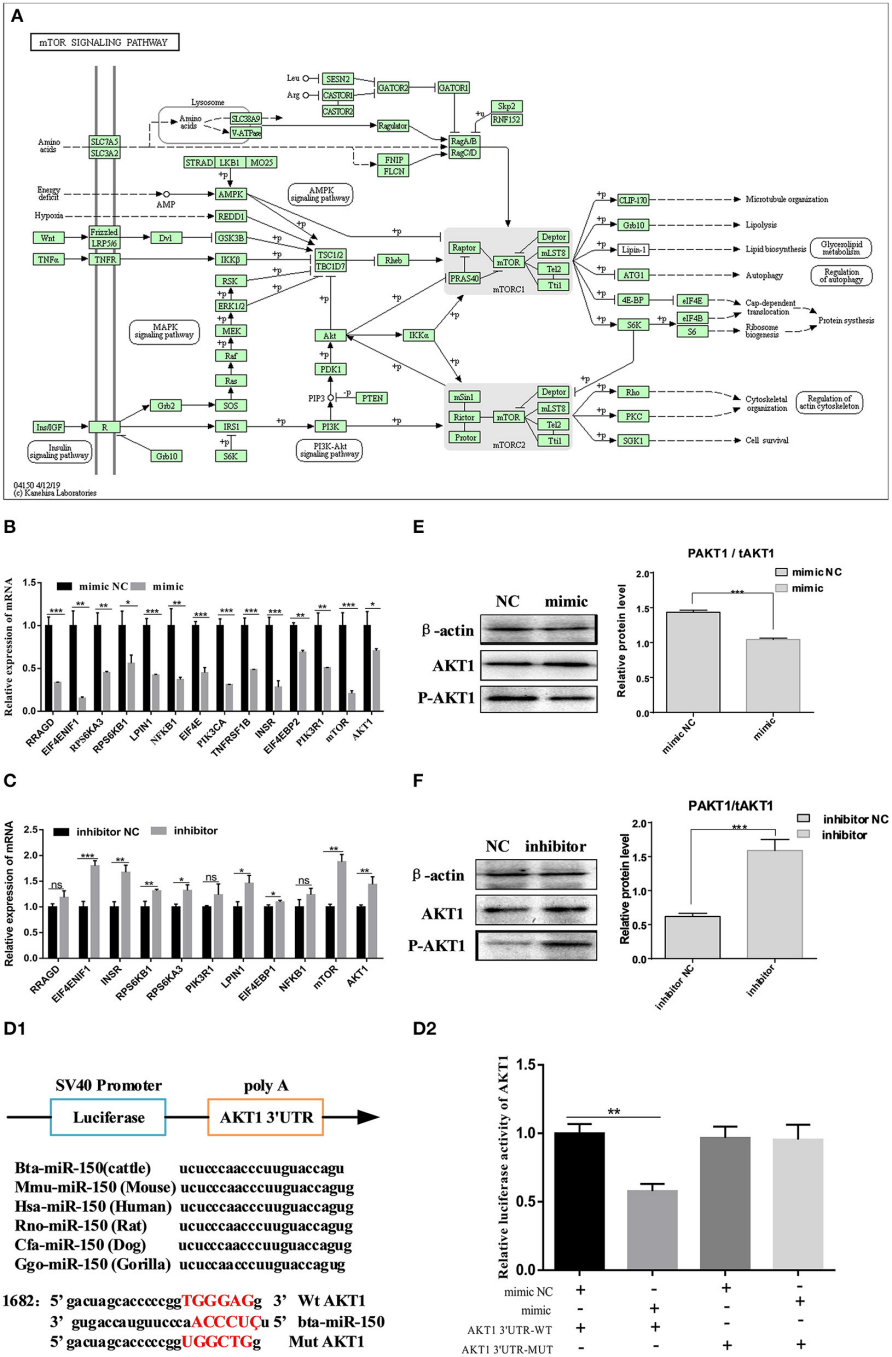
bta-miR-150 directly targets AKT1 and regulates adipocyte differentiation through mTOR pathway. **(A)** mTOR signaling pathway. **(B,C)** Changes in mRNA levels in the mTOR pathway on day 2 after incubation with the mimic, inhibitor, and NC. **(D1)** Construction of a double luciferase reporter vector; **(D2)** Changes in luciferase activity after co-transfection. **(E,F)** Western blots of Akt1 phosphorylation on day 2 of differentiation. Error bars, means ± SD. *n* = 3 replicates. *denotes significance according to Student's *t*-test, *p < 0.05, **p < 0.01, ***p < 0.001, and ****p < 0.0001.

We used targetscan7.1 to predict conserved target genes of miR-150 and the biological function of miR-150 during the development of bovine pre-adipocytes. We focused on the mTOR pathway based on the findings of the transcriptomic analysis (Figure 5A). Over 80% of the genes in the mTOR pathway were changed at the mRNA level by the miR-150 mimic or inhibitor (Figures 5B,C). Based on this and published information, we selected the adipocyte differentiation-related gene *AKT1* as a candidate target of bta-miR-150. We then constructed two luciferase reporter vectors for wild-type *AKT1* 3′UTR and mutant *AKT1* 3′UTR (Figure 5D1). Dual luciferase reporter assays revealed that *AKT1* is a target gene of bta-miR-150 (Figure 5D2). To further confirm the relationship between bta-miR-150 and *AKT1*, we measured *AKT1* expression during adipocyte differentiation at the mRNA and protein levels. The data indicated that bta-miR-150 promotes adipocyte differentiation by directly targeting *AKT1*. Western blot results showed that *AKT1* phosphorylation increased throughout differentiation up to a maximum of 1.0-fold after 48 h, compared with that at 0 h (Figures 5E,F). Taken together, these results implicate *AKT1* in fat differentiation in cattle and that *AKT1* is a target of bta-miR-150.

### si-AKT1 Can Inhibit Adipocyte Differentiation

**Figure 6:**
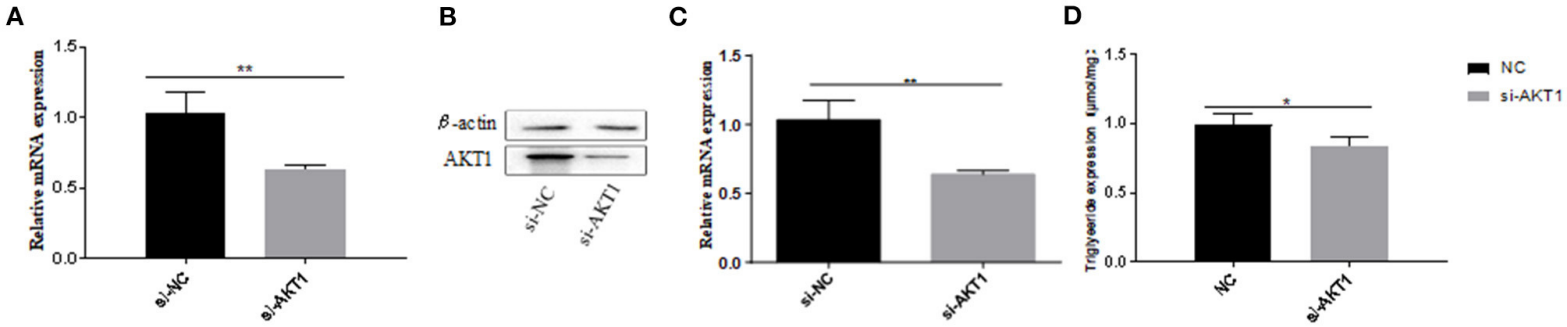
Effects of AKT1 on the metabolism of adipocytes in Qinchuan cattle. **(A)** The efficiency of si-AKT1 transfection; **(B,C)** The expression levels of AKT1 mRNA and protein in pre-adipocytes transfected with si-AKT1 and negative control cells were compared; **(D)** After si-AKT1 treatment, the triglyceride level in pre-adipocytes decreased. Error bars represent mean ± SD. *n* = 3 replicates. *denotes significance according to Student's *t*-test, *p < 0.05, **p < 0.01, and ***p < 0.001.

We used si-*AKT1* interference to determine the role of *AKT1* in pre-adipocytes from Qinchuan beef cattle. We determined the optimal siRNA concentration required to ensure high knockout efficiency before transfection (Figures 6A-C). We then showed that silencing the *AKT1* gene with 100 nM si-*AKT1* reduced triacylglycerol (TAG) levels in adipocyte precursors (Figure 6D), indicating that *AKT1* is a key transcription factor in pre-adipocytes from Qinchuan beef cattle.

## Discussion

We stained induced adipocytes with BODIPY on day 2 after differentiation and with Oil Red O on days 4 and 6 after differentiation. The number of lipid droplets was lower in the cells incubated with bta-miR-150 mimic than with mimic NC and higher in those incubated with miR-150 inhibitor than with inhibitor NC. These phenotypes remained consistent on days 2, 4, and 6 of the induction of bovine adipocyte differentiation. Further experiments were based on this phenotype. The results confirmed that miR-150 functions in bovine fat formation, indicating that it is a candidate for the regulation of adipogenesis in cattle. At present, the precise role of miR-150 in the regulation of adipocyte differentiation remains unclear. Many previous studies used low-throughput methods, which cannot sufficiently explain the molecular regulatory mechanisms through which miR-150 affects the formation of pre-adipocytes. Therefore, we used high-throughput RNA-seq to identify DEG in adipocytes with overexpressed or inhibited miR-150. The results of KEGG enrichment analysis of the DEG in adipocytes incubated with inhibitor and inhibitor NC on day 2 implicated the mTOR pathway (*p* = 0.0008), because most genes in the pathway were changed due to miR-150 inhibition, and most of these DEG (such as *INSR* (Cignarelli et al., 2019), *IGF1R* (Hancock et al., 2019), *RPS6KA1* (Song and Richard, 2015), and *NFKB1* (Akbar et al., 2015) were related to “fat metabolism.” To increase the richness of data analysis methods, we added the new WGCNA analysis (Langfelder and Horvath, 2008), which confirmed that among the multiple pathways enriched by DEG of five modules, the mTOR pathway was the most significant (*p* = 0.0092). Therefore, we verified that miR-150 mediates adipogenesis via the mTOR pathway. *AKT1* is part of the mTOR pathway and was implicated as a target of miR-150. Western blotting and qPCR findings confirmed that *AKT1* functions during adipogenesis.

Protein phosphorylation is a common and important post-translational modification (Humphrey et al., 2015). Approximately a third of cellular proteins are thought to be phosphorylated, and mTOR is a typical phosphorylation pathway (Rausch et al., 2019). Mammalian TOR plays an essential role in the regulation of cell growth and metabolism, such as responding to cell nutrients and growth signals, and it is associated with diabetes (Saxton and Sabatini, 2017). The seed sequence of miR-150 will be combined with the 3′ region sequence of *AKT1*. Qualcomm quantitative sequencing results have shown that when miR-150 is inhibited, insulin receptor substrates (IRS) are up-regulated, and receptor activation leads to the phosphorylation of key tyrosine residues on IRS proteins (Deng et al., 2020). After binding, the receptor activates p85 and recruits p10, which in turn catalyzes the generation of PI3P from PIP2 on the inner surface of the membrane. As the second messenger, PI3P further activates AKT and changes the phosphorylation level of *AKT* (Choi et al., 2010). *Akt* plays an important role in adipocyte differentiation (Xu and Liao, 2004). The “molecular switch” of BSTA can promote *AKT1* phosphorylation and promote adipocyte differentiation (Yao et al., 2013), whereas knocking out *Akt1* affects insulin-stimulated adipogenesis (Fischer-Posovszky et al., 2012). In addition, *Akt1/Akt2-*DKO mice have dyslipogenesis (Peng et al., 2003), and *AKT* deficiency in adipocytes can lead to severe lipodystrophy (Shearin et al., 2016). We found that miR-150 seems to inhibit bovine pre-adipocyte differentiation, which might inhibit the mTOR signaling pathway.

## Conclusions

Understanding the genetic basis of fat formation in beef cattle will support the breeding process to increase the intramuscular fat content of cattle. Our findings further validate and clarify the relationship between bta-miR-150 and bovine adipocyte differentiation; thus, overexpression of bta-miR-150 can inhibit adipocyte differentiation. Through RNA-seq sequencing data and WGCNA analysis methods, it was determined that the mTOR pathway, the signal pathway that plays a major role in bta-miR-150, affects bovine adipocytes' differentiation. This information paves the way for further research to elucidate the complex adipocyte differentiation and fat tissue production process.

## Data Availability Statement

The datasets presented in this study can be found in online repositories. The names of the repository/repositories and accession number(s) can be found below: NCBI SRA (accession: PRJNA684031).

## Ethics Statement

All animal procedures for experiments were approved by Committee of Experimental Animal Management (EAMC) at Northwest A&F University, China (protocol number: NWAFUCAST2018–168). Moreover, the use of experimental animals was carried out in accordance to the rules and guidelines of the organization and government. Written informed consent was obtained from the owners for the participation of their animals in this study.

## Author Contributions

XC, XM, SR, JW, XW, CL, and XY: conceptualization and data curation. XC, XM, SR, JW, CL, and XY: formal analysis. LZ: funding acquisition, project administration and supervision. XC, SR, CM, and XW: investigation. CL: methodology. JW and LZ: resources. XM, SR, and XY: software. XC, XM, CM, and XW: validation. XC: writing – original draft. SR: writing – review & editing. SS revised critically for content and grammar. All authors contributed to the article and approved the submitted version.

## Conflict of Interest

The authors declare that the research was conducted in the absence of any commercial or financial relationships that could be construed as a potential conflict of interest.
