# Corrigendum: Bovine Pre-adipocyte Adipogenesis Is Regulated by bta-miR-150 Through mTOR Signaling

**DOI:** 10.3389/fgene.2021.688741

**Published:** 2021-05-26

**Authors:** Xingyi Chen, Sayed Haidar Abbas Raza, Xinhao Ma, Jiangfang Wang, Xiaohui Wang, Chengcheng Liang, Xinran Yang, Chugang Mei, Syed Muhammad Suhail, Linsen Zan

**Affiliations:** ^1^College of Animal Science and Technology, Northwest A&F University, Xianyang, China; ^2^Department of Livestock Management, Breeding and Genetics, The University of Agriculture, Peshawar, Pakistan; ^3^National Beef Cattle Improvement Center, Northwest A&F University, Xianyang, China

**Keywords:** bta-miR-150, mTOR, RNA-seq, adipocyte differentiation, WGCNA

In the original article, there was a mistake in Figure 6A and 6D as published. The incorrect images were used for publication. The corrected Figure 6A and 6D appears below.

**Figure 6:**
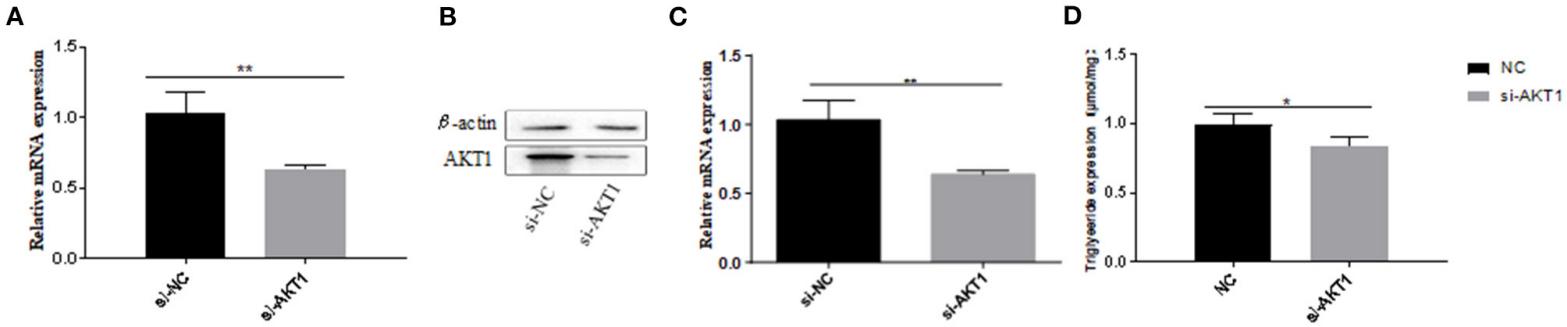
Effects of AKT1 on the metabolism of adipocytes in Qinchuan cattle. **(A)** The efficiency of si-AKT1 transfection; **(B,C)** The expression levels of AKT1 mRNA and protein in pre-adipocytes transfected with si-AKT1 and negative control cells were compared; **(D)** After si-AKT1 treatment, the triglyceride level in pre-adipocytes decreased. Error bars represent mean ± SD. *n* = 3 replicates. *denotes significance according to Student's *t*-test, *p < 0.05, **p < 0.01, and ***p < 0.001.

The authors apologize for this error and state that this does not change the scientific conclusions of the article in any way. The original article has been updated.

